# Urinary phthalate metabolite concentrations and adolescent sleep duration

**DOI:** 10.1097/EE9.0000000000000134

**Published:** 2021-02-18

**Authors:** Clara G. Sears, Joseph M. Braun

**Affiliations:** Department of Epidemiology, Brown University, Providence, Rhode Island

## Abstract

Some environmental chemicals, such as phthalates, are capable of disrupting endocrine function related to the development and regulation of sleep patterns. However, the contribution of phthalate exposure to inadequate sleep during adolescence is unknown. We aim to evaluate the association between phthalate exposure and short sleep duration during adolescence.

**Methods:**

We used data from participants 16–17 years of age in the 2005–2010 cycles of the National Health and Nutrition Examination Survey. Participants reported typical sleep duration during weekdays, which we categorized into short sleep duration (less than 8 hours per night) and adequate sleep duration (8 or more hours) based on consensus guidelines. We used weighted logistic regression to evaluate the association between log_10_-transformed urinary phthalate metabolite concentrations and odds of short sleep duration.

**Results:**

An interquartile range increase in di(2-ethylhexyl) phthalate metabolites, monocarboxynonyl phthalate, monocarboxyoctyl phthalate, and mono-isobutyl phthalate was associated with 1.48 (95% confidence interval [95% CI] = 1.02, 2.16), 1.64 (95% CI = 1.17, 2.32), 1.46 (95% CI = 0.99, 2.16), and 1.92 (95% CI = 1.08, 3.43) times the odds of short sleep duration, respectively. Furthermore, we observed monotonic dose-response relations between some phthalate metabolites and odds of short sleep duration.

**Conclusions:**

Our results suggest that higher urinary concentrations of several phthalate metabolites are associated with short sleep duration during adolescence. Understanding the contribution of modifiable environmental factors to inadequate sleep duration is of great public health importance because inadequate sleep can have substantial health implications.

**What this study adds**

Our results suggest that phthalate exposure may be associated with inadequate sleep duration during the adolescence. This area warrants further epidemiologic investigation in prospective studies given the vast number of adolescents reporting inadequate sleep and the importance of sleep to health.

## Introduction

In the United States, approximately 58% of middle schoolers and 73% of high schoolers did not get the recommended duration of sleep required for optimal health.^1^ Adolescents are susceptible to short sleep durations due to psychosocial influences and behavioral factors, as well as physiologic changes driven by gonadal hormones.^2–4^

Accumulating evidence suggests that phthalates—anthropogenic chemicals added to personal care and household products—affect the homeostasis or action of gonadal hormones, glucocorticoids, and thyroid hormones, potentially altering neurodevelopment and cardiometabolic health.^5–8^ Both early life and adolescent phthalate exposure could contribute to insufficient sleep during adolescence by disrupting neuronal circuitry and impeding maturation of hormonally mediated mechanisms that regulate development and sleep. However, we are not aware of any studies that have investigated if phthalates, or any endocrine-disrupting chemicals, impact sleep health during adolescence. Understanding the contribution of modifiable environmental factors, such as phthalate exposure, to sleep is of great public health importance because inadequate sleep can negatively impact behavior and increase obesity risk.^9,10^ We hypothesized that higher urinary phthalate metabolite concentrations would be associated with short sleep durations during adolescence.

## Methods

We investigated our hypothesis using data from 322 participants 16–17 years of age in the 2005–2010 cycles of the National Health and Nutrition Examination Survey (NHANES), a nationally representative cross-sectional study. We limited our analysis to these cycles because phthalates were assessed in a different subsample in 2011–012 compared with prior years and the sleep duration question was modified in 2015–2016. Participants 16 years and older were asked about sleep duration with the following question: “How much sleep do you usually get at night on weekdays or workdays?” with responses recorded in hours. We dichotomized average responses into short (less than 8 hours) and adequate (8 hours or more) sleep duration, based on recommendations for adolescents from the American Academy of Sleep Medicine.^11^

Participants provided urine samples and concentrations of phthalate metabolites (mono-ethyl phthalate [MEP], mono-benzyl phthalate [MBzP], mono (carboxynonyl) phthalate [MCNP], mono (carboxyoctyl) phthalate [MCOP], mono-n-butyl phthalate [MBP], mono-isobutyl phthalate [MIBP], and mono-(3-carboxypropyl) phthalate [MCPP], mono-(2-ethyl)-hexyl phthalate, mono-(2-ethyl-5-hydroxyhexyl) phthalate, mono-(2-ethyl-5-oxohexyl) phthalate, and mono-2-ethyl-5-carboxypentyl phthalate) were quantified using high performance liquid chromatography-electrospray ionization-tandem mass spectrometry. We calculated a measure of di(2-ethylhexyl) phthalate metabolites (ΣDEHP) by summing molar concentrations of the DEHP metabolites mono-(2-ethyl)-hexyl phthalate, mono-(2-ethyl-5-hydroxyhexyl) phthalate, mono-(2-ethyl-5-oxohexyl) phthalate, and mono-2-ethyl-5-carboxypentyl phthalate.

Based on NHANES Analytical Guidelines, we used weighted logistic regression to evaluate the association between log_10_-transformed urinary phthalate metabolite concentrations and odds of short sleep duration. We adjusted for log_10_-transformed urinary creatinine concentrations to account for urine dilution. We selected covariates a priori and included child race, age, and sex, NHANES wave, family poverty-to-income ratio, education level for the head of household, and children’s log_10_-transformed serum cotinine concentrations (a biomarker of tobacco smoke exposure). We assessed dose-response by categorizing phthalate metabolite concentrations into quartiles, assigning observations in each quartile the median concentration, and assessing the *P*-value for this term. We evaluated the robustness of our results by replicating our analysis in the sample of adolescents 16–18 years of age from the 2013 to 2014 NHANES cycle.

## Results

Approximately half (49%) of the 322 adolescents reported short sleep duration, averaging less than the recommended 8 hours per night. Demographic characteristics of adolescents with short sleep duration were similar to those reporting adequate sleep.

**Figure 1.**
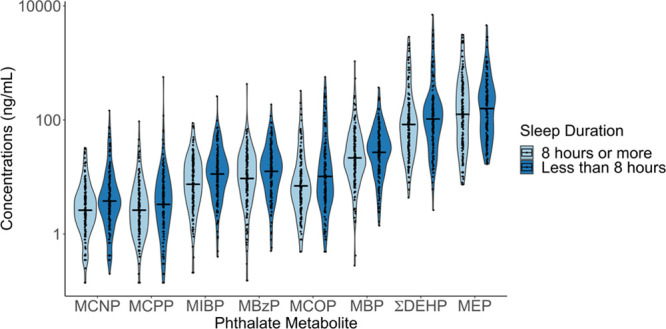
Distribution of log_10_-transformed urinary phthalate metabolite concentrations for adolescents (16–17 years of age) reporting adequate sleep (8 hours or more) compared with those reporting short sleep duration (less than 8 hours). Solid line indicates geometric mean of each distribution. Jittered dots are individual observations. Smoothed lines are density functions of urinary phthalate metabolite concentrations. ΣDEHP was calculated by dividing MEHP, MEHHP, MEOHP, MECPP by their respective molar mass, summing the molar metabolite concentrations, and then multiplying by the molar mass of MECPP to express the sum in units of ng/mL, Total participants = 322 (8 hours or more= 165; less than 8 hours= 157). MECPP, mono-2-ethyl-5-carboxypentyl phthalate; MEHHP, mono-(2-ethyl-5-hydroxyhexyl) phthalate; MEHP, mono-(2-ethyl)-hexyl phthalate; MEOHP, mono-(2-ethyl-5-oxohexyl) phthalate.

**Figure 2.**
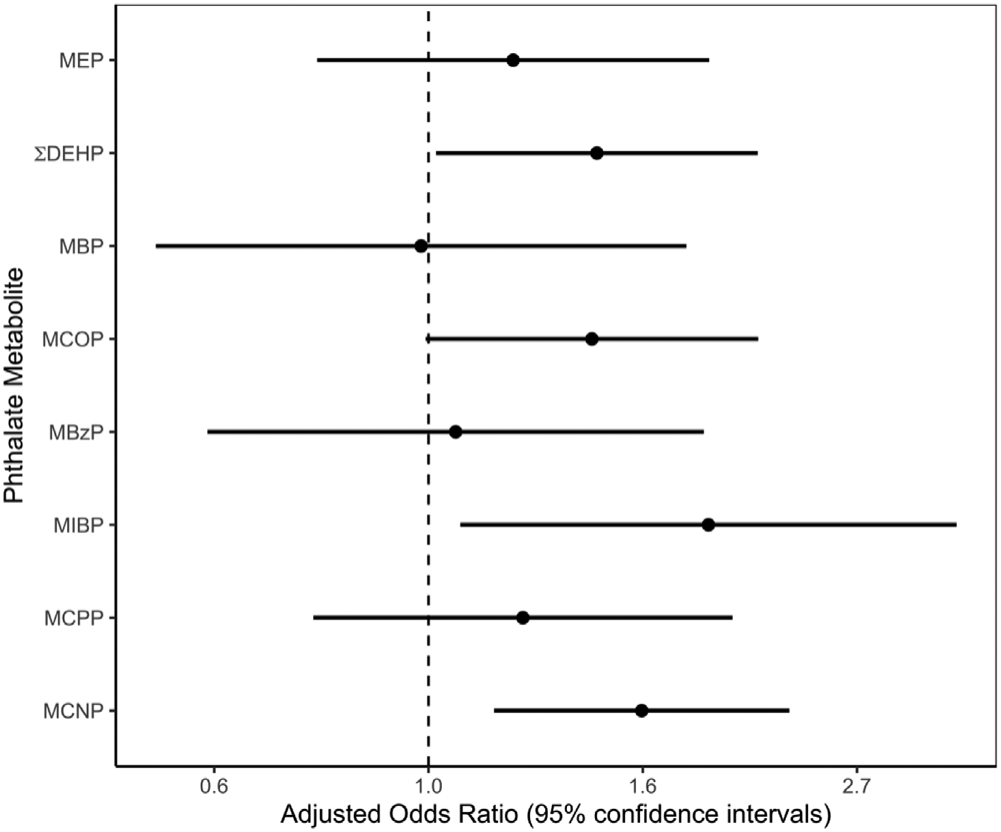
Adjusted odds of short sleep duration compared with adequate sleep duration associated with an interquartile range increase in log_10_-transformed urinary phthalate metabolite concentration among adolescents 16–17 years of age (NHANES, 2005–2010). Total participants = 322 (short sleep = 165; adequate sleep = 157). Adjusted for log_10_-transformed serum cotinine concentrations, race, age, sex, NHANES wave, family poverty-to-income ratio, and education level for the head of household.

Overall, adolescents with short sleep duration had higher geometric means of urinary phthalate metabolite concentrations compared with those getting adequate sleep (Figure 1). In adjusted models, higher urinary ΣDEHP, MCNP, MCOP, and MIBP concentrations were associated with higher odds of short sleep duration (Figure 2). An interquartile range increase in log_10_-transformed urinary ΣDEHP, MCNP, MCOP, and MIBP was associated with 1.48 (95% confidence interval [95% CI] = 1.02, 2.16), 1.64 (95% CI = 1.17, 2.32), 1.46 (95% CI = 0.99, 2.16), and 1.92 (95% CI = 1.08, 3.43) times the odds of short sleep duration, respectively. Furthermore, we observed positive monotonic dose-response relations of ΣDEHP (trend *P*-value = 0.020), MCNP (trend *P*-value = 0.020), and MIBP (trend *P*-value = 0.10) with higher odds of short sleep duration (Table 1).

**Table 1.** Odds of short sleep duration (less than 8 hours) compared with sleeping 8 or more hours associated with quartile of urinary phthalate metabolite concentrations among adolescents 16–17 years of age (NHANES, 2005–2010).

Phthalate metabolite	Short sleep (n)	Total (n)	Concentration, GM (Min, Max)	Adjusted, OR (95% CI)	Trend (*P*)
ΣDEHP					0.020
First	32	80	18.5 (2.6, 36.0)	Ref	
Second	37	81	59.6 (36.4, 87.9)	1.36 (0.73, 2.54)	
Third	42	77	122.7 (88.0, 184.1)	2.09 (0.89, 4.90)	
Fourth	46	84	511.7 (184.7, 7010.8)	2.76 (1.23, 6.19)	
MEP					0.46
First	29	82	25.9 (7.3, 48.0)	Ref	
Second	43	75	84.5 (48.6, 145.5)	2.60 (1.14, 5.96)	
Third	48	85	227.2 (146.8, 356.3)	1.33 (0.57, 3.11)	
Fourth	37	80	772.3 (360.6, 4568.6)	1.51 (0.66, 3.43)	
MBzP					0.94
First	33	79	2.3 (0.2, 5.4)	Ref	
Second	34	80	8.5 (5.6, 12.0)	0.57 (0.25, 1.31)	
Third	49	89	17.1 (12.0, 23.4)	0.88 (0.33, 2.35)	
Fourth	41	74	41.4 (23.5, 426.1)	0.84 (0.24, 2.93)	
MCNP					0.020
First	32	77	0.7 (0.1, 1.5)	Ref	
Second	34	80	2.3 (1.6, 3.1)	1.50 (0.49, 4.58)	
Third	40	82	4.1 (3.1, 5.6)	1.80 (0.69, 4.65)	
Fourth	51	83	12.6 (5.7, 146.1)	2.63 (1.13, 6.12)	
MCOP					0.11
First	31	77	1.7 (0.5, 3.6)	Ref	
Second	38	84	5.0 (3.6, 6.8)	0.80 (0.26, 2.39)	
Third	43	82	10.9 (6.9, 17.7)	1.36 (0.60, 3.11)	
Fourth	45	79	54.4 (17.8, 566.6)	1.85 (0.68, 5.02)	
MBP					0.55
First	37	81	5.9 (0.3, 12.6)	Ref	
Second	27	80	18.5 (12.8, 25.7)	0.46 (0.17, 1.28)	
Third	49	83	35.0 (25.9, 47.3)	1.73 (0.69, 4.49)	
Fourth	44	78	91.9 (47.4, 1063.6)	0.97 (0.28, 3.31)	
MIBP					0.10
First	30	83	2.2 (0.2, 4.7)	Ref	
Second	38	78	7.1 (4.8, 10.3)	1.06 (0.43, 2.61)	
Third	43	80	13.8 (10.4, 19.1)	2.29 (0.84, 6.28)	
Fourth	46	81	33.5 (19.2, 260.2)	2.54 (0.78, 8.29)	
MCPP					0.34
First	33	77	0.6 (0.1, 1.3)	Ref	
Second	34	81	1.9 (1.3, 2.9)	0.72 (0.28, 1.81)	
Third	39	79	4.1 (3.0, 6.0)	1.24 (0.45, 3.43)	
Fourth	51	85	13.0 (6.2, 564.0)	1.38 (0.42, 4.49)	

Adjusted model includes log_10_-transformed serum cotinine concentrations, race, age, sex, NHANES wave, family poverty-to-income ratio, and education level for the head of household

GM, geometric mean; max, maximum; min, minimum.

When we applied similarly adjusted models to adolescents 16–17 years of age from the 2013 to 2014 NHANES cycle, the odds ratios (ORs) for ΣDEHP (OR [95% CI] = 1.17 [0.63, 2.14]), MCNP (OR [95% CI] = 1.06 [0.65, 1.74]), MCOP (OR [95% CI] = 1.22 [0.61, 2.45]), and MIBP concentrations (OR [95% CI] = 0.90 [0.52, 1.56]) were smaller and less precise than the ORs for the previous cycles.

## Discussion

In this hypothesis-generating analysis, higher concentrations of some phthalate metabolites were cross-sectionally associated with shorter sleep duration during adolescence. We also found consistent, albeit attenuated, relations between ΣDEHP, MCNP, MCOP, and odds of short sleep duration when we replicated our analysis in a smaller sample from a subsequent NHANES cycle. However, in these sensitivity analyses, results for MIBP were inconsistent and should be interpreted with caution.

Previous research suggests that early life phthalate exposure is related to reduced mental and psychomotor development, poorer language development, and emotional problems^12–21^; these neurobehavioral characteristics may share biologic underpinnings with sleep health. Phthalates are a class of endocrine-disrupting chemicals commonly used as scent-retainers, emollients, and plasticizers in personal care products, food packaging, and building materials.^22–28^ Phthalates can be released from these products and are frequently ingested, dermally absorbed, or inhaled through indoor air.

Our results should be interpreted within the context of several limitations. Our analysis used self-reported sleep duration which is moderately correlated with estimates of sleep duration measured using actigraphy.^29^ Furthermore, healthy sleep requires more than adequate sleep duration. Sleep quantity is both influenced by and overlaps with dimensions of sleep quality, such as sleep efficiency and regularity in the timing of sleep periods. Further exploration of our research question with different types of sleep assessments and measures is warranted. Because sleep health can be influenced by a number of behaviors and environmental characteristics, such as light exposure, electronic screen use, environmental noise, and temperature, future research should also consider the possibility that these other factors may confound associations between phthalates and sleep outcomes.

Given that sleep questions were only administered to adolescents at age 16 years or older, our results may not be generalizable to younger adolescents who may have different sleep patterns. Additionally, the cross-sectional design of the NHANES precludes us from drawing conclusions about the directionality of these relations. It is possible that adolescents who stay up later and have short sleep durations may be more likely to have unhealthy dietary habits, and therefore consume more phthalate-containing foods.^30,31^ Previous research suggests that consuming more ultra-processed foods, for example, hamburgers and fries that are commonly sold at restaurants, is associated with higher urinary concentrations of MCNP, MCOP, and MCPP.^32^ Regardless of the direction, further investigation using a prospective study could potentially identify interventions to reduce phthalate exposure or improve adolescent sleep, both of which could ultimately lead to better health.
